# Next-generation antimicrobials: A review of phage lysins as precision weapons against drug-resistant pathogens

**DOI:** 10.1080/21505594.2025.2562634

**Published:** 2025-09-19

**Authors:** Xinge Cui, Luwei Chai, Yongkang Zhang, Yangwei Pan, Hongbing Liu, Xianlu Lei, Tao Le

**Affiliations:** College of Life Sciences, Chongqing Normal University, Chongqing, P. R. China

**Keywords:** Phage, phage lysin, antibiotic resistance, antimicrobial resistance, phage therapy, combination therapy

## Abstract

The escalating misuse of antibiotics has precipitated a dramatic rise in bacterial drug resistance, rendering conventional therapies increasingly ineffective. In this post-antibiotic era, phage lysins have emerged as a novel class of antimicrobial agents, attracting significant attention for their precise targeting of drug-resistant pathogens. Despite promising preclinical results – highlighting their rapid bactericidal efficacy and synergistic potential with other antimicrobials – the clinical translation of lysins remains limited by unresolved challenges related to efficacy, safety, pharmacokinetics, and dosing optimization. This review provides a comprehensive overview of lysin research advancements, discusses diverse application strategies, and critically evaluates therapeutic outcomes from animal models and early clinical trials. Additionally, it addresses the key obstacles impeding lysin development and proposes practical solutions and future research directions to unlock the full clinical potential of this innovative antimicrobial strategy.

## HIGHLIGHTS

- Phage lysins offer a targeted strategy to combat drug-resistant bacterial infections.
- Engineered lysins improve antibacterial efficacy through fusion with other molecules.
- Combining lysins with antibiotics enhances treatment and reduces resistance risks.
- Challenges in stability, delivery, and immunogenicity limit lysins’ clinical application.
- Future research should focus on optimizing lysin design and large-scale production.

## Graphical abstract

**Figure:**
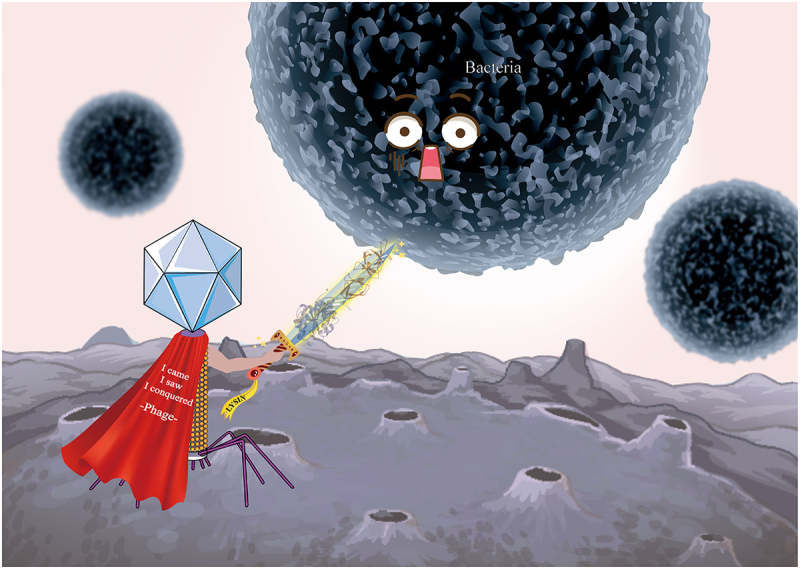
This review explores the challenges and prospects of phage lysins in the post-antibiotic era, focusing on their use as precision weapons against drug-resistant pathogens and clinical translation.

## Introduction

Since Alexander Fleming discovered penicillin in 1928, antibiotics have been hailed as a revolutionary means to treat bacterial infections. However, widespread antibiotic use has led to a rapid rise in bacterial resistance and the emergence of superbugs. Multidrug-resistant (MDR) bacteria – defined as pathogens resistant to at least one antibiotic in three or more classes owing to the presence of resistance determinants such as extended-spectrum β-lactamases and mobile polymyxin resistance (MCR) enzymes – pose a severe threat to global public health. Guo et al. discovered for the first time that up to 96% (1,168 out of 1,217) of Aeromonas strains harbored at least one protein belonging to the MCR family. This discovery enables a deeper understanding of the MDR mechanism [1]. In the post-antibiotic era, the pace of new antibiotic discovery has lagged behind the escalating problem of resistance, rendering even common infections potentially fatal and underscoring the urgent need for alternative therapeutic strategies.

Phage therapy, which originated in 1919 with the treatment of bacillary dysentery [2], was largely abandoned with the advent of antibiotics but has regained interest in light of increasing drug resistance [3,4]. Phages offer several advantages over traditional antibiotics, including high specificity, rapid bactericidal action, and extensive natural distribution. Moreover, their capacity to co-evolve with bacteria gives phage therapy a distinct advantage in combating MDR bacteria [5,6]. Nevertheless, the narrow host range of phages remains a significant limitation. Consequently, the discovery of new phages, such as VB_ValC_WD615, vB_PaeM_PS3, Kpph1, Kpph9, LPCS28, and LPEK22 [7–11], which display powerful lytic activity, a wide host range, and high environmental tolerance, still remains a matter that merits attention.

In contrast, phage-encoded lysins have demonstrated efficacy against a broad range of drug-resistant bacteria. While most lysins are typically species- or genus-specific, some exhibit activity against multiple Gram-positive bacteria, or even across both Gram-positive and Gram-negative groups, thereby effectively broadening their lytic spectrum. Unlike intact phage particles, lysins specifically degrade the peptidoglycan layer, a vital and highly conserved component of the bacterial cell wall, thereby reducing the likelihood of resistance emergence [12].

Consequently, lysins have emerged as highly promising protein-based alternatives to traditional antibiotics. Similar to phages, lysins can rapidly kill target bacteria within seconds without disturbing the normal microbial flora [13]. Furthermore, lysins are more readily accepted as protein therapeutics and can be produced at scale using both prokaryotic and eukaryotic expression systems. Their bactericidal activity is independent of the bacterial growth phase, allowing them to lyse both actively growing and dormant cells. In addition, lysins can act synergistically with other antimicrobial agents, further enhancing their therapeutic potential.

This review presents a comprehensive overview of lysin research, including structural diversity and mechanisms of action. We summarize current application strategies and clinical prospects, critically evaluate data from animal models and clinical trials, and discuss pivotal issues such as dosing regimens, efficacy, and safety. Finally, we address the main obstacles limiting lysin development, propose practical solutions, and outline future directions for these novel antimicrobials.

## History of lysin

Phages are viruses that infect bacteria and are widely distributed in soil, oceans, and human habitats. They were independently discovered by Twort and d’herelle [3,14–16]. It was not until 1958 that Jacob et al. first reported that phages could encode proteins that lysed bacteria, lysins [17], and demonstrated that they played an important role in the phage infection cycle. In 1959, Freimer et al. reported for the first time that purified lysins exhibited bactericidal ability, and since then, an increasing number of lysins have been identified and investigated for various applications [18]. Starting in the 1990s, García and colleagues demonstrated in a series of studies that pneumococcal lysins evolved by modular exchange, a study that supported the hypothesis that the C-terminal structural domain of lysin has a substrate recognition role [19–21]. In 2001, Nelson et al. conducted the first prophylactic and therapeutic experiments using in vitro-purified lysin in animal models. Their results confirmed that lysins were capable of killing host bacteria both in vitro and in vivo and of protecting mice from infection [22].

**Figure 1.**
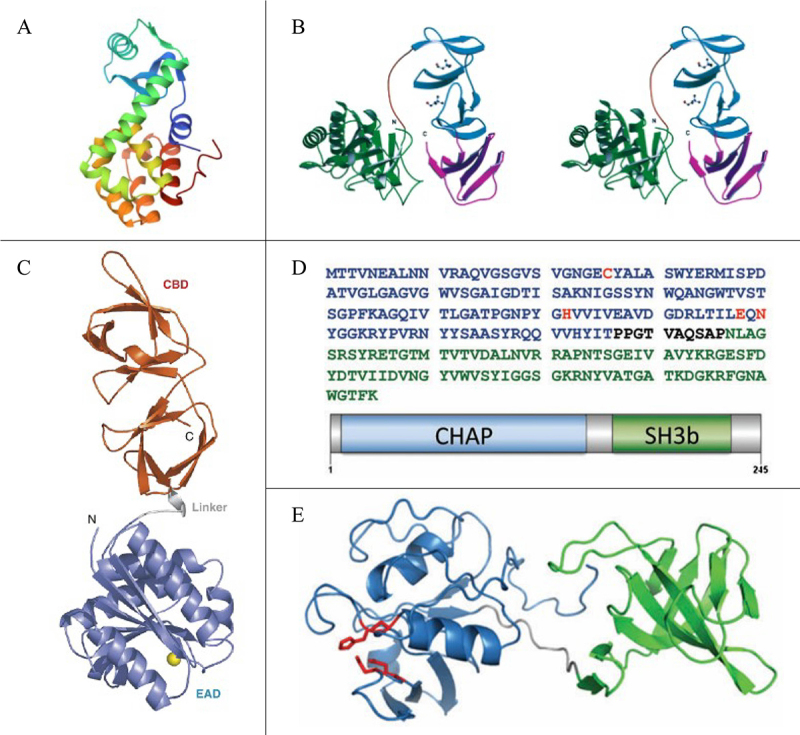
Evolution of phage lysins with protein sequence alignment and structural modeling. (A) Refined structure of T4 phage lysozyme at 1.7 Å resolution (PDB ID: 2LZM) [23]. Copyright © 1987, Elsevier Ltd. (B) Stereo view of Cpl-1 with color-coded domains: catalytic N-terminal (green), linker (orange), CI (cyan), and CII (magenta); choline molecules shown as ball-and-stick [24]. Copyright © 2003, cell Press, Elsevier Ltd. (C) Crystal structure of PlyPSA. The enzymatic domain (blue), linker (grey), and cell wall-binding domain (red) are shown; catalytic Zn^2 +^ (yellow sphere) (PDB ID: 1XOV) [25]. Copyright © 2006, Elsevier Ltd. (D) Schematic of CF-301, showing the CHAP domain (blue), SH3b domain (green), and active-site residues (red), identified by homology to PDB 2K3A [26]. Copyright © 2013, the Author, Oxford University Press. (E) Ribbon model of CF-301 built using I-TASSER, with PDBs 2K3A and 1R77 as templates. Active-site residues shown; color scheme as in (D). Model rendered in PyMOL [26]. Copyright © 2013, the Author, Oxford University Press.

In 1987, the structure of T4 lysozyme was refined to a resolution of 1.7 Å for the first time, marking a significant milestone in lysin research (Figure 1(A)) [23]. Through X-ray crystallography, this study revealed the detailed three-dimensional structure of T4 lysozyme, clarifying its active site and substrate-binding region and laying a critical foundation for subsequent investigations. As structural studies advanced, the term “lysozyme” gradually evolved into “lysin” to better reflect its diverse mechanisms of action and functions.

In 2003, Hermoso et al. reported the first fully resolved structure of a lysin containing a choline-binding module – Cpl-1, a pneumococcal phage lysin – along with its full-length crystal structures in both free and choline-bound states (PDB ID: 1H09, 1OBA) (Figure 1(B)). Cpl-1 consists of an N-terminal and a C-terminal domain, responsible for catalytic hydrolysis and substrate binding, respectively. The hydrolysis and binding domains together adopt a hairpin-like structure, with the C-terminal binding domain composed of two distinct subdomains, each containing six consecutive repeats [24]. Three years later, Korndörfer published the second lysin with a fully modular structure, PlyPSA (PDB ID: 1XOV) (Figure 1(C)). PlyPSA originates from the temperate Listeria monocytogenes phage PSA, and its structural model, determined by X-ray crystallography, revealed a complete domain organization. Notably, the C-terminal domain of PlyPSA exhibits a novel folding pattern, forming internal structural repeats without significant sequence homology [25].

**Figure 2.**
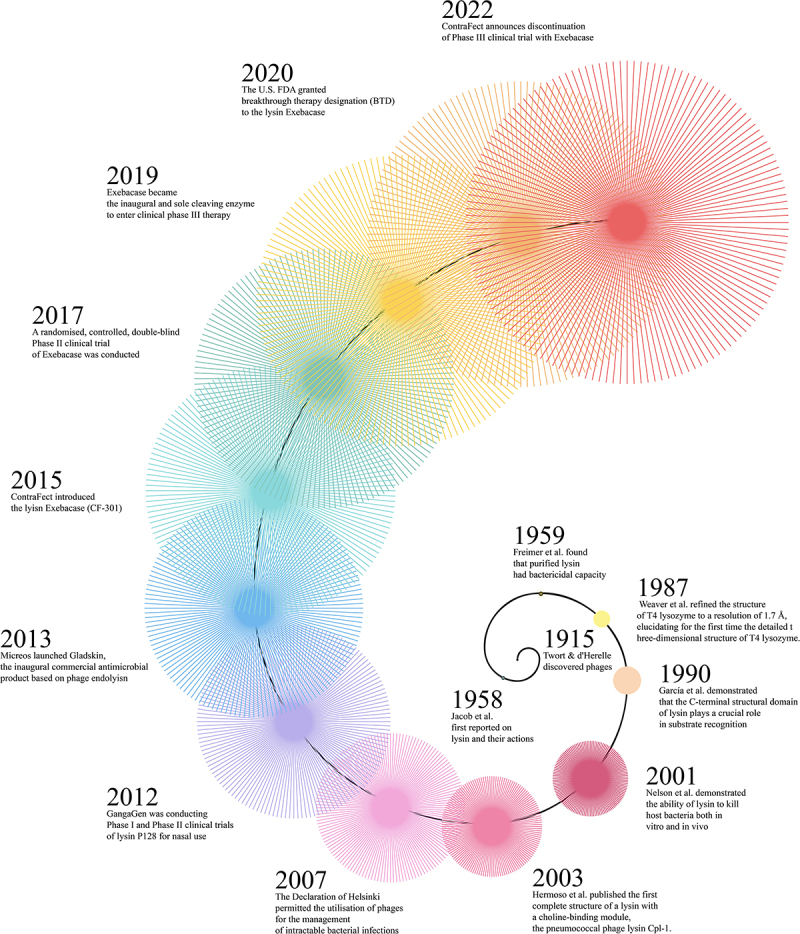
Timeline of phage lysin discovery and development.

As the structural resolution of lysins has improved and the functions of their domains have become better understood, significant progress has been made in their clinical applications. Since 2007, phage therapy for refractory bacterial infections has been authorized under §37 of the Declaration of Helsinki, established by the World Medical Association [27], accelerating the pace of clinical trials involving phages and their derived enzymes. In 2012, Ganggen, an Indian company, developed a lysate against *Staphylococcus aureus* (including methicillin-resistant *S. aureus*, MRSA) lysin P128 for nasal use, with associated combined Phase I and Phase II clinical trials after testing in prior animal models (Clinicaltrials.gov.NCT01746654). In 2013, Micreos launched Gladskin, the world’s first commercial antimicrobial product based on the phage-derived lysin, with the main active ingredient being the lysin enzyme Staphefekt SA.100 to combat atopic dermatitis caused by *S. aureus*, for which clinical trials have been conducted (Clinicaltrials.gov.NCT02840955) [27]. Among all lysins tested clinically, Exebacase (CF-301), developed by ContraFect, is undoubtedly the most prominent. Phase I clinical trials of the lysin Exebacase started in 2015 (Clinicaltrials.gov.NCT02439359) showed that intravenous infusion of CF-301 (0.04–0.4 mg/kg over 2 hours) was well-tolerated without notable safety signals (Figure 1(D,E)). In 2017, a randomized, controlled, double-blind Phase II clinical trial of Exebacase (Clinicaltrials.gov.NCT03163446) was conducted, revealing that the cure rate in the Exebacase-treated group exceeded that of the antibiotic group by over 40% [28]. Thus, in 2020, the U.S. Food and Drug Administration (FDA) granted breakthrough drug status (BTD) to the lysin Exebacase [29]. In 2019, Exebacase became the first and only lysin to enter clinical phase III treatment (Clinicaltrials.gov.NCT04160468) [30]. However, in 2022, ContraFect announced the termination of this trial, as it ultimately failed to meet the study endpoint. Although post hoc analysis showed no statistically significant enhancement in day-14 clinical response for MRSA bacteremia/endocarditis patients receiving Exebacase plus antibiotics, the study nonetheless provides valuable insights for future clinical development of lysins [31], as reflected in the development timeline (Figure 2).

## Diversity of lysins

### Structure

Phage lysins exhibit remarkable diversity, with their bactericidal mechanisms closely linked to their structural characteristics [17]. The structural composition of lysins differs between Gram-positive and Gram-negative bacteria, primarily due to differences in their cell wall architecture. Phage lysins targeting Gram-positive bacteria typically display a “dual-domain” organization, consisting of an N-terminal enzymatically active domain (EAD) and a C-terminal cell wall-binding domain (CBD) [32], connected by a short peptide linker. In some cases, lysins also contain an amidase module, which can significantly enhance their lytic activity [33]. The binding domain specifically recognizes and attaches to the bacterial cell wall, facilitating the lysis of intact cells in the surrounding medium, while the catalytic domain cleaves molecular bonds within the bacterial peptidoglycan [34]. Building on this modular structure, subsequent studies have classified lysins based on the cleavage site of the catalytic domain within peptidoglycan. According to this classification, lysins can be divided into five major groups: (1) acetylcytidylic acidases, which cleave the β-1,4 glycosidic bond linking N-acetylcytidylic acid to N-acetylglucosamine; (2) transglycosidases, which cleave at the same site as acetylcytidylic acidases but through a different mechanism; (3) aminoglucosidases, which cleave the β-1,4 glycosidic bond between GlcNAc and MurNAc; (4) amidases, which cleave the amide bond between MurNAc and L-alanine; (5) endopeptidases, which cleave peptide bonds within peptide chains.

**Figure 3.**
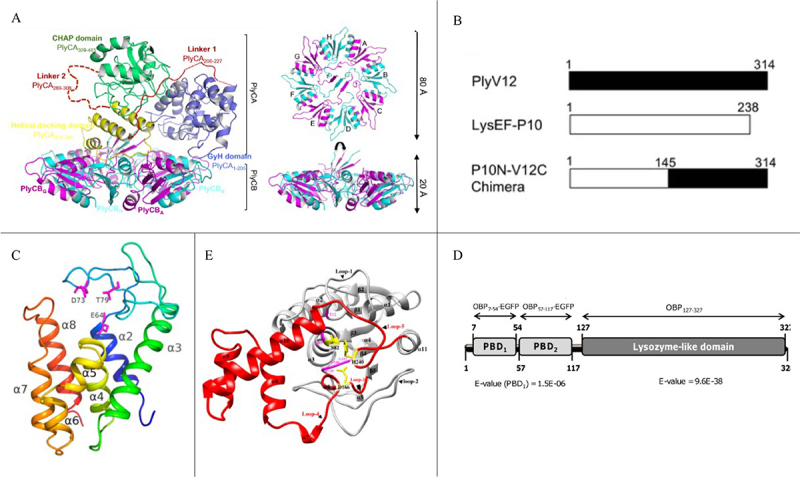
Structural diversity of phage lysins in different host bacteria. (A) For gram-positive bacteria, the structure consists of a single PlyCA catalytic subunit assembled with eight PlyCB molecules. In this panel, the PlyCB cell wall-binding domain is alternately colored in magenta and cyan and is labeled from a to H [36]. Copyright © freely available online through the PNAS open access option. (B) For gram-positive bacteria, this schematic illustrates both the parental lysins and their chimeric recombinants created through domain swapping. The N-terminal region functions as the catalytic domain, while the C-terminal portion serves as the cell wall-binding domain [37]. Copyright © 2020 Binte Muhammad Jai, Dam, Tay, Koh, Loo, Kline, and Goh. (C) For gram-negative bacteria, the crystal structure of AcLys was resolved at 1.2 Å resolution. The AcLys monomer displays an overall α-helical Fold typical of the lysozyme family, with a color gradient from blue at the N-terminus to red at the C-terminus [38]. Copyright © 1996–2025 MDPI (Basel, Switzerland). (D) For gram-negative bacteria, a schematic representation indicates that the predicted N-terminal peptidoglycan-binding domains (PBD) are shown in light grey, while the C-terminal catalytic domains (resembling lysozyme-like or goose-type lysozyme domains) are depicted in dark grey [39]. Copyright © 2012 Walmagh et al. (E) For mycobacteria, the crystal structure of LysB-D29 is presented, highlighting secondary structure features such as the linker domain (in red), catalytic triad residues (in yellow), as well as the oxyanion hole and GNP residues (in pink); the remainder of the protein is shown in gray [40]. Copyright © 1996–2025 MDPI (Basel, Switzerland).

Phage lysins derived from Gram-positive bacteria typically range from 25 to 40 kDa in molecular weight (for comparison, antibiotics typically range from 0.3 to 1.6 kDa) [35]. Nearly all lysins are encoded by a single gene, with the notable exception of the PlyC, a lysin from the C1 phage of group C *Streptococcus*, which consists of two separate genes: PlyCA and PlyCB (Figure 3(A)). These two genes encode the binding domain, comprising eight subunits that self-assemble into a donut-shaped structure [41]. PlyCA encodes the catalytic domain, with two PlyCA non-covalently associated with the PlyCB complex, forming the unique architecture of this lysin [36].

When comparing lysin sequences within the same enzyme class, it is typically observed that the EAD region is highly conserved, exhibiting a high degree of amino acid sequence homology, while the CBD region is highly variable. This suggests that lysins are specifically adapted to target their hosts. This characteristic ensures that the enzyme, which induces cell lysis, can spill out and target nearby potential bacterial hosts. For instance, in a study of *Clostridium difficile*, the CBD of the phage lysin was found to be homologous to the cell wall-associated module of bacterial cell wall proteins, likely acquired during phage-host coevolution. Subsequent experiments revealed that the CBD of lysin is essential for anchoring the enzyme to post-lysis cell wall remnants, a property that limits enzyme diffusion. As a result, selecting CBD-free lysins may provide a viable strategy for treating *C. difficile* infections [42].

Although most previous chimeric lysin constructs failed to achieve species specificity, the modular architecture of Gram-positive bacterial lysins provides the potential for flexible combinations of EAD and CBD. Jai et al. demonstrated that when a broad-spectrum CBD from various lysin profiles was fused with a narrow-spectrum EAD to construct the chimeric lysin P10N-V12C (Figure 3(B)), the broad-spectrum CBD activity was overridden by the narrow-spectrum EAD, which played a dominant role in the final lysin profile. As a result, the enzyme exhibited species specificity against *Enterococcus faecalis* and *Staphylococcus*. This finding suggests that, while CBD is often regarded as the primary determinant of the cleavage profile, EAD also plays a critical role in modulating species selectivity. These results provide new insights into designing chimeric lysins with targeted specificity through the strategic selection of EADs [37].

Gram-negative bacteria pose a distinct challenge. The outer membrane (OM) of these bacteria acts as a natural barrier, preventing lysins from entering the cell interior. As a result, most phage lysins targeting Gram-negative bacteria are single-domain enzymes that lack a CBD [43–45]. These lysins are generally globular, around 15–20 kDa in size, and consist solely of an EAD. They also carry a positively charged group at the C-terminus, which enables the enzyme to penetrate or disrupt the OM, as well as interact with and degrade the peptidoglycan layer, ultimately leading to bacterial lysis [38,46]. For instance, the C-terminal α-helical structure of AcLys (Figure 3(C)) works synergistically with its positively charged groups to further enhance OM penetration [38]. Although most Gram-negative lysins are single-domain, notable exceptions exist. KZ144 and EL188, both derived from *Pseudomonas aeruginosa*, exhibit a modular architecture comprising an N-terminal substrate-binding domain and a C-terminal catalytic module. These binding domains contain conserved sequences, a feature previously only observed in Gram-positive bacterial lysins but in reverse order, allowing these enzymes to exhibit high affinity and broad-spectrum activity against Gram-negative pathogens [47,48]. In addition, the *Pseudomonas fluorescens* phage lysin OBPgp279 contains both N-terminal CBD and C-terminal lysozyme structural domains, displaying modular features akin to those of Gram-positive bacteria (Figure 3(D)). Its lysing activity is 160 times greater than that of T4 lysozyme [39]. Finally, OM permeability in Gram-negative bacteria is influenced by molecular structure and LPS diversity. For instance, Abp013 shows strong lytic activity against *Acinetobacter baumannii* and *Klebsiella pneumoniae*, but is ineffective against *P. aeruginosa*. After ruling out differences in the catalytic domain, this selectivity was attributed to variations in the C-terminal cationic region of Abp013 [49].

The cell wall structure of *Mycobacterium* is more complex than that of both Gram-positive and Gram-negative bacteria. It comprises a mycobacteriophage-arabinogalactan-PG complex, where PG is covalently linked to arabinogalactan, which is further esterified by long α-branched, β-hydroxy fatty acids (mycolic acids), forming a robust lipid barrier. This unique architecture confers *Mycobacterium* with substantial resistance to lysis [50]. To overcome this barrier, phages infecting *Mycobacterium* have evolved two lysins. LysA is capable of degrading the PG layer, which is essential for cell wall degradation, while LysB cleaves the mycolic acid, glycolipid, and lipid arabinomannan network, targeting the branched ester bonds of the arabinogalactan layer [51–53]. Notably, LysB is a phage-specific enzyme and has been more frequently reported than LysA [54]. Studies have shown that LysA and LysB act synergistically, with LysB increasing the vulnerability of *Mycobacterium* to LysA. Although LysA alone can inhibit cell growth, it is not sufficient for complete lysis. In contrast, the synergistic effect of LysB plays a critical role in achieving effective lysis [55]. Recent research on the D29 phage has provided promising evidence supporting the use of LysB as a novel therapeutic agent (Figure 3(E)) [40,56].

### Mechanisms of action

The mechanism of action of phage lysins can be categorized into two types: endolysis and ectolysis. Endolysis occurs when a phage infects a host bacterium, commandeers its transcription and translation machinery to produce viral proteins, and subsequently synthesizes lysins to facilitate cell lysis upon virion assembly completion. These lysins then degrade the bacterial cell wall, leading to lysis from the inside out. In contrast, ectolysis refers to the external application of phage lysins to bacterial cells. In this case, lysins directly cleave the peptidoglycan layer from the outside, resulting in structural collapse of the cell wall, osmotic imbalance, and eventual bacterial death.

**Figure 4.**
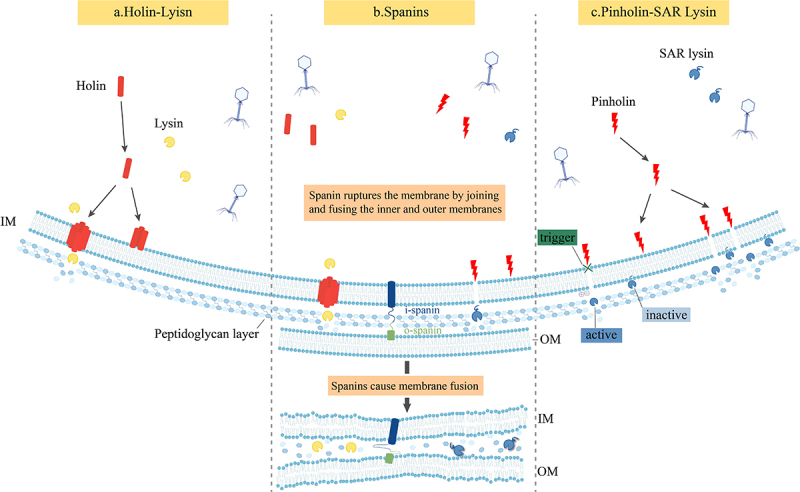
Three inside-out mechanisms of phage lysis.

Phage-mediated inside-out lysis primarily relies on the synergistic action of lysin, holin, or pinholin, and auxiliary lysis proteins such as spanin. In Gram-positive bacteria, the classical Holin-Lysin system predominates: holin forms pores in the bacterial inner membrane, allowing lysin to access the peptidoglycan layer, where it degrades the cell wall, ultimately leading to structural disintegration and bacterial lysis [57]. This mechanism is widely observed in Gram-positive bacteria. In contrast, Gram-negative bacteria possess an additional barrier – a lipopolysaccharide-rich OM – beyond the peptidoglycan layer, making lysis more complex. Their phage lysis process requires not only the Holin-Lysin system but also the cooperative action of Spanin proteins. In this process, Lysin first degrades the peptidoglycan layer, while Spanin is responsible for disrupting the OM, ultimately completing lysis through the formation of the Holin-Lysin-Spanin lysis system. Spanin consists of two key components: i-Spanin, an inner membrane protein, and o-Spanin, an OM protein. These two subunits work in concert to mediate the fusion of the inner and OMs, eventually leading to OM collapse and bacterial lysis. Additionally, certain dsRNA phages (e.g. λ21) utilize the Pinholin-SAR Lysin lysis system [58]. In this system, Pinholin forms multiple small pores in the inner membrane rather than creating a single large perforation. The accumulation of these small pores induces inner membrane depolarization, triggering a conformational change in the membrane-anchored SAR Lysin. This structural shift releases SAR Lysin into the peptidoglycan layer, where it enzymatically degrades the bacterial cell wall, ultimately completing the lysis process [59], as illustrated by the inside-out lytic mechanism (Figure 4) and supported by representative lysins listed in Table 1.

**Table 1.** Comparison of phage lysis mechanisms.

Mechanism	Mode of Action	Key Proteins	Steps	Target Bacteria	Additional Features
Holin-Lysin	Holin forms large pores in the inner membrane, allowing Lysin to access the periplasm and degrade the peptidoglycan layer, leading to bacterial lysis.	Holin (controls lysis timing), Lysin (degrades peptidoglycan).	(1) Holin forms large pores in the inner membrane; (2) Lysin crosses the membrane through these pores; (3) Lysin degrades the peptidoglycan layer, causing cell lysis.	Primarily Gram-positive bacteria; ineffective against Gram-negative bacteria without additional mechanisms.	(1) Well-suited for single-membrane bacteria (mainly Gram-positive); (2) Precise timing control via Holin acting as a molecular clock; (3) Ineffective for Gram-negative bacteria, as the outer membrane remains intact.
Holin-Lysin-Spanin	An extended version of the Holin-Lysin system, incorporating Spanin to disrupt the outer membrane of Gram-negative bacteria, ensuring complete lysis.	Holin (pore formation), Lysin (peptidoglycan degradation), Spanin (outer membrane disruption).	(1) Holin forms pores in the inner membrane; (2) Lysin degrades peptidoglycan; (3) Spanin facilitates inner and outer membrane fusion, leading to outer membrane collapse.	Gram-negative bacteria.	(1) Requires Spanin to rupture the outer membrane (without it, lysis is incomplete); (2) Spanin consists of i-Spanin (inner membrane) and o-Spanin (outer membrane), working together to destabilize the outer membrane; (3) Holin forms large pores in a single-step event.
Pinholin-SAR Lysin	Pinholin forms multiple small pores, leading to inner membrane depolarization, which triggers the activation and release of SAR Lysin to degrade the peptidoglycan layer.	Pinholin (small pore formation, depolarization), SAR Lysin (peptidoglycan degradation).	(1) Pinholin forms small pores; (2) Inner membrane depolarization; (3) SAR Lysin undergoes conformational change and detaches from the membrane; (4) SAR Lysin degrades peptidoglycan, causing lysis.	Certain phages (e.g. φX174), capable of lysing Gram-negative bacteria.	(1) Pinholin forms small pores rather than large ones, leading to gradual accumulation; (2) SAR Lysin activation depends on membrane depolarization (remains inactive if the membrane is not depolarized); (3) Effective for Gram-negative bacteria, but may not require Spanin.

**Figure 5.**
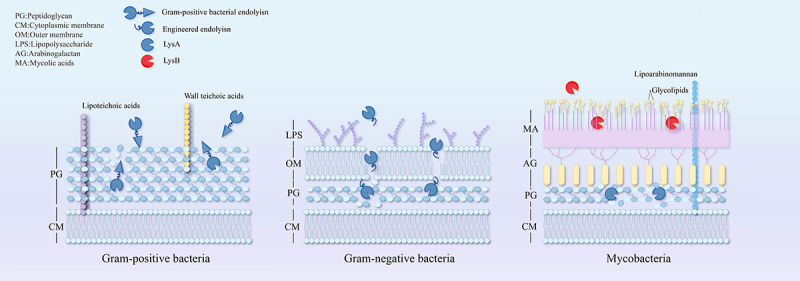
Outside-in lytic action of phage lysins on gram-positive, gram-negative, and mycobacteria.

Autolysis mediated by phage lysin refers to bacterial lysis triggered by the direct, exogenous action of certain phage-derived enzymes on the bacterial cell wall [52,60]. In Gram-positive bacteria, phage lysins can rapidly degrade the peptidoglycan layer upon contact, leading to cell wall rupture and bacterial death. Representative examples include LysGH15 [61], Cpl-1 [62] and ClyJ [63]. In contrast, Gram-negative bacteria possess an outer membrane (OM) that acts as a barrier, preventing lysins from reaching the underlying peptidoglycan. To circumvent this obstacle, several strategies have been employed (1): co-administration with OM-permeabilizing agents such as polylysine, polymyxin B, or disodium ethylenediaminetetraacetate (EDTA) (2); engineering chimeric lysins by fusing them with membrane-penetrating peptides; and (3) delivery via OM-permeabilizing vectors. Representative lysins employing these strategies include PlyPa03 [38], recombinant LysSS [64], and Lysep3 [65], as illustrated in Figure 5.

## Potential applications of lysin

**Figure 6.**
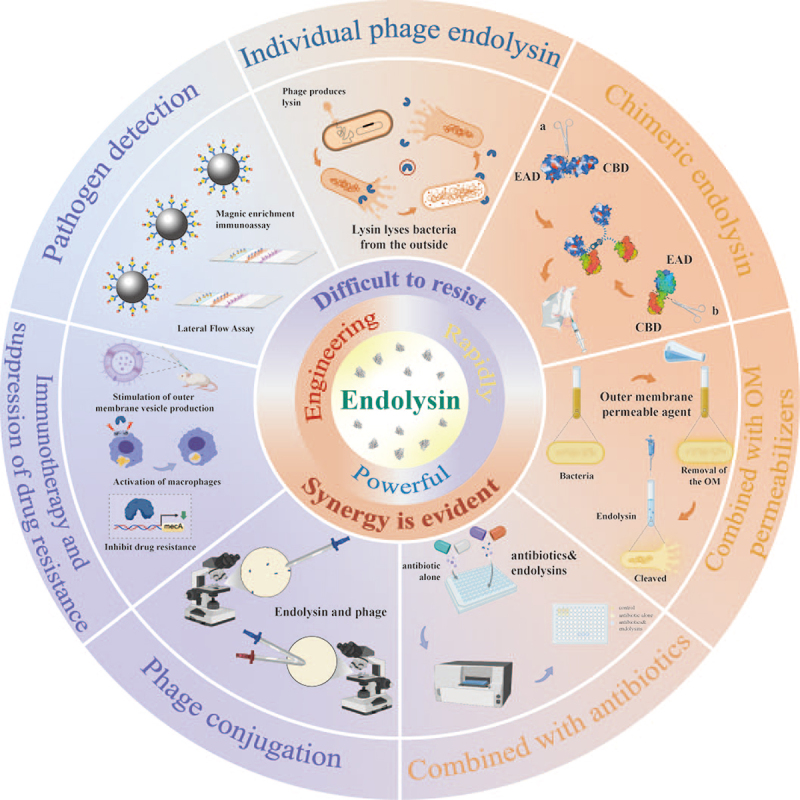
Diverse application strategies of phage lysins.

Lysin has rapidly gained traction across diverse fields, including – but not limited to – clinical medicine, animal husbandry, and food safety. This widespread application is largely due to its potent and rapid bacteriolytic activity, which rivals that of antibiotics, while demonstrating a lower likelihood of inducing bacterial resistance. Rather than focusing solely on application contexts, this section highlights the specific strategic modalities through which lysins can be effectively employed. As illustrated in Figure 6, lysins can be harnessed via seven key approaches, each tailored to different clinical or technological settings.

### Individual phage lysins

Phage lysin has emerged as a promising therapeutic agent in the post-antibiotic era, offering distinct advantages over conventional antibiotics. They exhibit potent and rapid bactericidal activity, a markedly lower propensity to induce bacterial resistance, and high specificity toward pathogenic bacteria without disrupting the native microbiota [66]. Extensive studies have shown that recombinant lysins can effectively degrade bacterial biofilms, significantly reduce bacterial loads, alleviate clinical symptoms of infection, and ultimately improve host survival rates.

Lysins have demonstrated superior efficacy compared to antibiotics in targeting MDR pathogens. Li et al. successfully expressed the lysin LysP53, which showed potent in vitro activity against a broad spectrum of antibiotic-resistant Gram-negative bacteria, including *A. baumannii*, *P. aeruginosa*, *K. pneumoniae*, and *Escherichia coli*. In a murine burn infection model caused by *A. baumannii*, a single intraperitoneal dose of 14 μg LysP53 (57.6 μM) resulted in significantly greater bacterial reduction than either 4 μg minocycline (874 μM; *p* < 0.05) or the buffer control (*p* < 0.001), achieving a 3-log decrease in bacterial load [67]. Xi et al. further demonstrated the potential of the lysin AVPL as a therapeutic agent for mastitis caused by *Aerococcus viridans*. In vitro, 2 μg/mL of AVPL reduced *Mycobacterium oxysporum* in milk samples by approximately 2-log_10_ within 1 hour, exhibiting excellent bactericidal activity. In an *A. viridans*-induced mouse mastitis model, a single 25-μg dose of AVPL significantly reduced bacterial loads in the mammary glands (~2-log_10_), alleviated mastitis-related pathological symptoms, and decreased inflammatory cytokine levels (TNF-α, IL-1β, and IL-6) in mammary tissues [68]. In addition, AVPL displayed broad-spectrum bactericidal activity against various serotypes of *Streptococcus suis*. In vitro, 300 μg/mL AVPL achieved a 4–4.5-log₁₀ reduction in bacterial counts within 1 hour. AVPL not only inhibited biofilm formation for up to 48 hours but also effectively disrupted pre-formed biofilms. In a bacteremia mouse model induced by a multidrug-resistant *S. suis* type 2 (SS2) strain SC19, a single 300-μg dose of AVPL provided complete protection against lethal infection, significantly reduced bacterial burden across multiple organs, and alleviated systemic inflammation and histopathological damage [69]. These findings underscore AVPL’s potential as a novel antimicrobial agent for treating diverse bacterial infections.

The application of individual lysins has expanded beyond laboratory and animal models. Xu et al. engineered a novel lysin, PlyEc2, which exhibited potent bactericidal activity against major Gram-negative pathogens, including *E. coli*, *Salmonella*, *Shigella*, *Fusobacterium*, and *Pseudomonas*. In vitro, PlyEc2 achieved complete sterilization, reducing bacterial titers by more than 5-log₁₀. In a longleaf lettuce leaf model, PlyEc2 eliminated 99.7% of Shiga toxin-producing *E. coli* (STEC) O157:H7 contamination without compromising the visual appearance or texture of the lettuce [70]. Similarly, Pastagia et al. developed a ClyS-based topical ointment for the treatment of *S. aureus* skin colonization and infection in a mouse model. Compared to the standard topical antimicrobial mupirocin, ClyS significantly reduced methicillin-sensitive *S. aureus* (MSSA) and MRSA loads, with its antibacterial activity remaining unaffected by varying antibody titers [71]. Collectively, these studies underscore the broad potential of lysins as stand-alone antimicrobial agents, demonstrating their consistent and robust bactericidal efficacy across diverse application settings.

### Chimeric enzymes

As previously discussed, lysins typically possess a modular architecture, consisting primarily of the EAD and CBD. This structural modularity, coupled with the independent functionality of each domain, offers significant potential for engineering. The functional domains of natural lysins can be selectively removed, recombined, and fused with other regulatory molecules, enabling the design of bioengineered lysins with tailored functionalities.

The most crucial step in constructing chimeric enzymes is the meticulous selection of the primary candidates. Roehrig et al. utilized a chimeric PGH library that fuses the EAD and CBD of PGH, both known for their high activity and unique structural domain arrangement (CHAP_M23_SH3b and M23_CHAP_SH3b) [72]. Enzyme activity was validated in human serum, ultimately leading to the selection of MEndoB. In vivo experiments showed that MEndoB effectively treated zebrafish larvae infected with *S. aureus*. Further investigations in a mouse infection model revealed that MEndoB significantly reduced *S. aureus* loads and TNF-α levels in a dose-dependent manner, resulting in improved animal survival [73]. Páramo et al. combined the EAD of the lysin Skl with the CBD of the pneumococcal lysin LytA to successfully generate the chimeric lysin QSLA, which exhibits enhanced antimicrobial activity. QSLA markedly improved bacterial killing, with efficacy increased by two orders of magnitude or more, and expanded the substrate range to include resistant strains and emerging pneumococci. In contrast, the reciprocal chimera QLAS, which contains the LytA EAD and Skl CBD, showed no bactericidal activity against any tested strains [74].

In addition to the diverse domain combinations intrinsic to lysins, these enzymes can also be engineered by fusing with antimicrobial peptides (AMPs) or other bioactive agents. Kim et al. fused LysC02, a lysin derived from phage ΦC02, with nine different AMPs to generate LysC02-AMP. This fusion triggers the physical destruction of bacterial cells by disrupting the OM and increasing internal swelling pressure. The results indicated that LysC02-AMP significantly improved the survival rate of *Galleria mellonella* larvae infected with *Cronobacter sakazakii*, and substantially reduced the presence of *C. sakazakii* in both food and food-contact surface models [75]. Nie et al. employed computational design to construct four new chimeric lysins (P361, P362, P371, and P372), which combine Salmonella phage lysin with the novel antimicrobial peptide LeuA-P. The combination of P362, P372, and potassium sorbate reduced microbial counts in contaminated chilled chicken by more than 3 log CFU/g and extended shelf life by 7 days [76].

Recombinant engineering of phage lysins offers an effective strategy for expanding lytic spectra, improving environmental stability, and overcoming bacterial resistance mutations. To optimize bacteriolytic activity and broaden target coverage, recombinant lysins can be rationally designed by fusing functional domains from multiple phage lysins. Such engineered constructs may exhibit enhanced enzymatic potency, an expanded range of antimicrobial activity, or improved specificity toward particular bacterial species.

### Combination with external membrane permeabilisers

Gram-negative bacteria possess a thick OM that significantly impairs the effectiveness of lysin. To overcome this, peptidoglycan (PG) substrates capable of altering OM permeability are considered critical for facilitating lysin penetration into Gram-negative bacteria [77,78]. Commonly used outer membrane permeabilising agents (OMPs) are EDTA [79], chloroform (CHCl3) [80], citric acid [81], polylysine [82], and cationic dendritic polymers [83]. These OMPs often include chelating agents, with EDTA being a key example. EDTA functions by binding to divalent cations, removing them from their binding sites, and leading to OM disruption [84]. Similarly, weak organic acids such as citric acid exhibit chelating properties, with their permeabilizing effectiveness dependent on their protonation state. For instance, trisodium citrate shows minimal permeabilizing activity at neutral pH, whereas citric acid acidifies the medium, enhancing OM disruption [85]. Briers et al. investigated the effectiveness of OM permeabilization in various antibiotic-resistant strains of *P. aeruginosa* using EDTA, citric acid, polylysine, and polymyxin B nonapeptide (PMBN) [86].

Similar to other phage-encoded endolysins, LysKP213 cannot directly lyse Gram-negative bacteria due to the barrier function of the OM. To overcome this, Zhu et al. pretreated *K. pneumoniae*, *A. baumannii*, *P. aeruginosa*, and *E. coli* with EDTA to remove the OM before adding LysKP213. Their results demonstrated that LysKP213 effectively lysed the majority of the tested Gram-negative strains [87]. Wang et al. expressed the lysin LysP6 in prokaryotic cells, and it was shown to lyse *Salmonella* of different serotypes, as well as the standard strain *E. coli* ATCC25922, after the bacterial OM was treated with chloroform. Combined with the outer membrane permeabilizer EDTA, LysP6 significantly inhibited *Salmonella* growth within 24 hours, and its host range was notably broader than that of phage P6 [79]. OMPs not only improve the bactericidal efficacy of lysins but also expand their lytic spectrum. For instance, lysin ABgp46 exhibited antibacterial activity only against *A. baumannii* in the absence of OMPs. However, when citric and malic acids were added, the antibacterial activity of ABgp46 against *A. baumannii* was enhanced, and it also exhibited lytic activity against *P. aeruginosa* and *Salmonella typhimurium* [88]. Unlike Gram-positive bacteria, the OM of Gram-negative bacteria often prevents lysin from accessing the peptidoglycan layer, thereby limiting its activity. The selection of appropriate OMPs can effectively expose peptidoglycan and facilitate lysin action. Nevertheless, this approach requires the addition of exogenous agents to the enzymatic system, and their safety must be thoroughly evaluated for practical applications [89].

### Combination with antibiotics

It has previously been demonstrated that the combination of lysins and OMPs represents a promising strategy, enabling lysins to target Gram-negative bacteria and broaden their host spectrum. In addition, antibiotics, as traditional antimicrobial agents, have shown significant potential in synergizing with phages and lysins to combat various pathogenic bacteria. Studies have indicated that combining phages and antibiotics can enhance antibiotic efficacy against MDR bacteria by prolonging or even restoring the antibiotic effect on specific strains [90]. Furthermore, antibiotics can potentiate the antimicrobial effects of lysins and help mitigate the development of bacterial resistance. Consequently, the combined application of lysins and antibiotics has attracted increasing scientific interest [91,92].

Mucomycin, a polycationic peptide antibiotic, competitively binds to lipopolysaccharides (LPS), displacing divalent cations such as Ca^2 +^ and Mg^2 +^, thereby destabilizing the OM [93]. In a study by Zhang et al., visfatin was combined with LysAB2 to investigate their synergistic effects against MDR *A. baumannii* (MDR-AB2), with a focus on evaluating the stability of the combination and its potential cytotoxicity under varying conditions. The results revealed that a threshold concentration of mucomycin (0.2–0.8 μM) was effective in permeabilizing the outer membrane, allowing lysin to reach the peptidoglycan layer and perform its cleavage activity. The effective threshold concentration was influenced mainly by the density of LPS molecules on the OM. Above this concentration, LysAB2 synergized with fucoxanthin at levels as low as 0.31 μM. Notably, for the first time, it was shown that LysAB2 facilitated disruption of the cytoplasmic membrane of mucins by degrading the peptidoglycan layer, which subsequently increased reactive oxygen species (ROS) levels within bacterial cells and enhanced mucin’s bactericidal effects. Considering lysin’s biofilm-disrupting ability, the mucin-lysin combination effectively eradicated established biofilms. The synergistic antimicrobial activity of this combination was further validated in vivo using the *Galleria mellonella* infection model. Zhang et al. thereby elucidated the mechanism underlying the synergy between visfatin and LysAB2: by disrupting multiple bacterial membrane layers, this combined therapy not only enhanced bactericidal efficacy but also reduced potential nephrotoxicity and neurotoxicity associated with lower visfatin dosages [94].

Sosa et al. employed a DAIR mouse model of prosthetic joint infection (PJI) to evaluate the efficacy of PlySs2 combined with vancomycin in reducing periprosthetic bacterial burden. Their findings demonstrated that this combination therapy achieved substantial reductions in CFU counts, achieving a 92% reduction on the implant surface and an 88% reduction in periprosthetic tissue. The study emphasized that vancomycin’s effectiveness is largely dependent on the metabolic state of bacteria, making it more effective against planktonic cells. Conversely, PlySs2 specifically targets *S. aureus* biofilms, diminishing bacterial loads within biofilms and enhancing bacterial susceptibility to antibiotics. In this murine DAIR model for established PJI, the PlySs2-vancomycin combination not only enabled a reduction in the required antibiotic concentration but also effectively disrupted biofilms, significantly lowering bacterial loads in both periprosthetic tissue and implant surfaces. While vancomycin is conventionally used against MRSA infections, Sosa et al. highlighted its synergistic potential with PlySs2 in treating MSSA, the predominant causative agent of PJI [95]. In a separate experiment, Watson et al. selected Exebacase – the first lysin evaluated in a Phase II clinical trial – to perform in vitro synergy experiments with 12 different antibiotics. The results revealed that in most interactions, fractional inhibitory concentration index (FICI) values ≤ 0.5 indicated synergy. No notable differences in synergy were found between MSSA and MRSA strains. Specifically, Exebacase demonstrated the strongest synergistic effect when combined with antibiotics targeting the cell membrane (e.g. daptomycin, vancomycin, nafcillin, benzoxiline, cefazolin, and travancin), particularly against strains resistant to these antibiotics. This synergistic effect likely reflects a “re-sensitization effect,” in which the β-lactam MIC of MRSA is reduced to within the CLSI susceptibility range. The enhanced synergy may result from lysin-induced disruption of the bacterial cell wall, facilitating improved antibiotic penetration to target sites and/or increasing antibiotic efficacy [96].

### Conjugation with phage

While the synergistic interaction between lysins and antibiotics has been well documented, enabling enhanced therapeutic outcomes with reduced antibiotic dosages, accumulating evidence suggests that certain antibiotics may adversely affect phage therapy. Specifically, antibiotics that inhibit nucleic acid or protein synthesis can indirectly impair phage replication, thereby compromising therapeutic efficacy [97]. The extent of this negative interaction is influenced by both the duration of antibiotic exposure and its concentration [97,98]. Moreover, the use of antibiotics inherently carries the risk of promoting resistance development. In contrast, combining phage therapy with lysins offers a promising alternative that can circumvent the major limitations associated with phage-antibiotic co-therapy. This approach not only avoids interference with phage replication but also leverages the complementary mechanisms of action of phages and lysins to enhance antibacterial efficacy while minimizing the risk of resistance.

Duarte et al. conducted an insightful study to evaluate the combined application of the lysin CHAPSH3b and the virulent phage phiIPLA-RODI. Their results demonstrated a significantly greater reduction in viable *S. aureus* cell counts when both agents were applied together. Time-kill curves and confocal microscopy observations indicated that CHAPSH3b rapidly reduced the bacterial population within 7 hours of initial exposure, followed by phage adsorption and bacterial lysis, thereby limiting bacterial regeneration. Moreover, at least 90% of phage-resistant strains were susceptible to CHAPSH3b, suggesting that the lysin may help delay the emergence of phage resistance during treatment. The combination of CHAPSH3b and phiIPLA-RODI also resulted in a faster short-term reduction of bacterial populations in a model of isolated porcine skin wound infection. Additionally, lysin assists phages in biofilm removal by lysing part of the bacterial population and increasing the phage-to-bacteria ratio. Beyond this effect, CHAPSH3b may potentiate phage activity by targeting phage-resistant subpopulations within biofilms and disrupting the extracellular matrix, ultimately increasing bacterial susceptibility to phage infection. These findings highlight multiple complementary roles of lysins in phage-based antimicrobial strategies [99].

Overall, accumulating evidence supports the concept that combining phage-derived lysins with bacteriophages represents a promising and innovative antimicrobial strategy. Looking ahead, the development of diverse phage – lysin combination therapies is anticipated to offer effective solutions against the persistent and evolving challenge of bacterial drug resistance.

### Immunotherapy and drug resistance suppression

As research into lysins continues to expand, their additional functional roles – particularly in immunotherapy and in mitigating bacterial drug resistance – are emerging as significant areas of interest. These novel applications provide added value and represent promising directions for future phage-based therapeutic strategies.

Outer membrane vesicles (OMVs) are nanoscale (20–250 nm) spherical proteoliposomes released by Gram-negative bacteria during growth or infection. Although widely applied in vaccine development, their low natural yields present a significant bottleneck [100–102]. Li et al. discovered that the highly active *A. baumannii* phage lysin, LysP53, could stimulate OMV production upon interaction with *A. baumannii*, *E. coli*, and *Salmonella*. Compared to naturally produced OMVs, LysP53-induced OMVs (LOMVs) exhibited better results, including improved homogeneity, significantly lower endotoxin content, and increased protein production. Data from a mouse model of *A. baumannii* infection demonstrated that LOMVs provided protection equal to or better than natural OMVs in both intramuscular and intranasal immunity [103]. In another study, Yang et al. were the first to describe the application of lysin-derived V12CBD in preventing *S. aureus* infection. Specifically, binding of V12CBD to *S. aureus* significantly suppressed the expression of several key virulence genes, with reductions ranging from 2.4 to 23.4-fold. More importantly, V12CBD activated macrophages via the NF-κB pathway, enhancing their phagocytosis of *S. aureus*. In both therapeutic and prophylactic models, V12CBD provided significant protection against MRSA infection in mice [104]. Schuch et al. demonstrated that co-administration of CF-301 and daptomycin delayed the emergence of daptomycin resistance in S. aureus MW2 during a 26-day serial passaging experiment [26]. Similarly, Singh et al. reported that repeated exposure to Ply187 over 10 generations did not induce resistance in *S. aureus*, further highlighting the potential of lysins as sustainable antimicrobial agents [105].

### Pathogen detection

The CBDs of most lysins guide the catalytic domain to its specific substrates in the bacterial cell wall by means of non-covalent interactions with carbohydrate ligands. It is estimated that a single bacterial cell may present over 10^7^ CBD binding sites, underscoring the remarkable specificity and affinity of CBDs [106]. These properties make CBDs highly promising candidates for the development of innovative bacterial detection tools. Yu et al. developed a rapid and straightforward method for detecting *S. aureus* in milk by integrating immunomagnetic separation with lysin CBD. This method enabled the detection of as few as 400 CFU of *S. aureus* in 100 μL of milk within just 1.5 hours [107]. In a separate study, Liu et al. utilized the C54A mutant of LysGH15—engineered to abolish lytic activity while retaining high binding affinity – and immobilized it on a BLI biosensor for *S. aureus* detection. The biosensor distinguished live from dead bacteria within 12 minutes, achieving an impressive detection limit as low as 13 CFU/mL, highlighting its strong potential for practical application [108].

## Therapeutic effects of lysin in vivo

Several lysins have been isolated and characterized for the treatment of MDR bacteria, including the predominant Gram-positive pathogen, *S. aureus*, as well as major Gram-negative bacteria such as *A. baumannii*, *K. pneumoniae*, *P. aeruginosa*, and *E. coli*. These organisms are classified as “critical priority” by the WHO due to their high virulence and multidrug resistance, highlighting the urgent need for novel therapeutic approaches. As previously discussed, various lysin-based therapeutic strategies have demonstrated promising efficacy in animal models. With lysin therapy advancing into clinical trials, this section examines specific dosage regimens, therapeutic outcomes, and safety evaluations observed in vivo, as detailed in Table 2. Overall, lysins have shown strong antimicrobial activity and favorable safety profiles when administered at appropriate doses. However, substantial challenges remain before lysin-based therapies can be widely translated into clinical practice.

**Table 2.** Summary of preclinical efficacy of phage-derived lysin.

Lysin	Target bacteria	Animal infection model	Diseases	Dose regimen	Result	Ref.
LysAB2	*A. baumannii*	*G. mellonella* larvae infection model	Systemic	A single dose of 10 mg/larvae LysAB2 alone or with colistin (0.028–7.2 μg/larvae), 0.5 h post-infection via last right proleg injection.	Low colistin dose (0.056 μg/larvae): no bacterial load reduction. High dose (3.6 μg/larvae): ~2 log CFU/ml bacteria eliminated with LysAB2.	Zhang et al. [94]
LysAB2-KWK	*A. baumannii*	*G. mellonella* larvae infection model	Systemic	A single dose of 5 μg/larva LysAB2-KWK, 0.5 h post-infection via last right proleg injection	Survival: 60% at 24 h, 50% stable at 48–96 h. Significantly higher than the control group.	Chen et al. [109]
AVPL	*A. viridans*	Mouse infection model	Mastitis	After 1 h of infection, mice were delivered with 12.5, 25, or 50 μg/gland of AVPL.	25 μg/gland AVPL: ~2 Log_10_ bacterial load reduction, improved pathology, decreased cytokines (TNF-α, IL-1β, IL-6).	Xi et al. [68]
MEndoB	*S. aureus*	Zebrafish larvae & Mouse infection model	Systemic	Zebrafish: 4 ng/fish MEndoB, 2 h post-infection via injection. Mice: 10, 1, or 0.1 mg/kg MEndoB, 2 h post-infection via intraperitoneal injection.	Zebrafish: 100% survival at 21 h. Mice: survival at 48 h was 90%, 80%, 40% for 10, 1, 0.1 mg/kg groups, respectively.	Roehrig et al. [73]
AVPL	*S. suis*	Mouse infection model	Bacteremia	300, 150, or 50 μg/mouse AVPL, 1 h post-infection via intraperitoneal injection.	Survival after 7 days: 100%, 70%, 10% for 300, 150, 50 μg/mouse groups, respectively.	Xi et al. [69]
PlyB	*B. cereus*	Mouse infection model	Endophthalmitis and keratitis	Endophthalmitis: 1 µL of 420 µg/mL PlyB via intravitreal injection at 2 h post-infection. Keratitis: one drop of 420 µg/mL PlyB every hour for five doses starting 2 h post-infection via topical application.	Endophthalmitis: bacterial load reduced by 5 log_10_ at 4 h post-treatment. Keratitis: > 4 log_10_ reduction in CFUs at 7 h post-infection.	Mursalin et al.[110]
PlyKp104	*K. pneumoniae*	Mouse infection model	Skin infections	300 μg PlyKp104 via topical application at 3 h post-infection.	>2 logs reduction in bacterial load at 3 h post-treatment compared to control.	Euler et al. [111]
Ply1228	*S. suis*	Mouse infection model	Bacteremia	200 μg/mouse Ply1228 via intraperitoneal injection at 1 h post-infection.	Significant protection (*p* < 0.0001), reduced bacterial loads in blood and organs, alleviated inflammation, and reduced histopathological damage.	Wang et al. [112]
LSVT-1701	MRSA	Rabbit infection model	Aortic valve infective endocarditis	Multiple regimens of LSVT-1701 (32.5 or 50 mg/kg i.v., single or multiple doses) + daptomycin 4 mg/kg i.v. QD for 4 days starting 24 h post-infection.	LSVT-1701 + daptomycin significantly reduced MRSA in target tissues. Four daily doses sterilized all target tissues.	Huang et al. [113]
ClyJ-3 m	*S. pneumoniae*	Mouse infection model	Bacteremia	Single intraperitoneal dose of 2.32 or 1.16 μg/mouse, 1 h post-infection.	2.32 μg/mouse dose provided 70% protection.	Luo et al. [114]
PlySs2	MRSA	Mouse infection model	Prosthetic joint infection	Vancomycin (110 mg/kg, SC, q12h) + PlySs2 (2.5 mg/kg, IP, q24h), days 5–10.	Combination reduced CFUs by 92% on implants and 88% in periprosthetic tissue.	Sosa et al. [95]
LysSS	*A. baumannii* & *P. aeruginosa*	BALB/c mice	Systemic	Intraperitoneal infection with 10^9^ CFU, divided into six groups: control, infection control, 125 μg LysSS safety, 500 μg LysSS safety, 125 μg LysSS treatment, and 500 μg LysSS treatment.	125 μg LysSS treatment group had the highest survival (40% at 4 d). 500 μg LysSS resulted in 100% mortality within 1 d, earlier than infection control (2 d). No deaths in control or safety groups over 6 days.	Kim et al. [64]
ClyJ-3	*S. pneumoniae*	Mouse infection model	Systemic	Intraperitoneal infection with 8 × 10^7^ CFU/mouse NS26.	100% protection at 100 μg/mouse, 83% at 50 μg/mouse, and 30% at 2 μg/mouse.	Yang et al. [63]
Vplys60	*V. parahaemolyticus*	Artemia franciscana infection model	Systemic	Artemia challenged with *V.parahaemolyticus* and Pichia expressing Vplys60 (1.29 × 10^7^ cells/ml)	Treatment resulted in a significant increase in survival with a 70% reduction in mortality.	Srinivasan et al. [115]
LysB	*M. ulcerans*	Mouse infection model	Footpad mouse model of *M. ulcerans*	Treatment was initiated when footpad swelling reached 2.7 mm. Two subcutaneous injections of 50 μg LysB in PBS were administered at 10 and 13 days post-infection; controls received PBS.	LysB treatment prevented further bacterial multiplication, achieving a 1-log reduction compared to untreated controls at day 16 post-infection.	Fraga et al. [116]
Ply5218	*S. suis*	Piglet infection model	Systemic	Purified lysin was administered as three doses of 150 µg/kg (total 450 µg/kg) at 6, 24, and 48 h post-challenge.	Bacterial load in blood was significantly reduced, with no bacteria detected at 7 days post-infection.	Wang et al. [117]
PlyPa03	*P. aeruginosa*	Mouse infection model	Skin & lung infections	Infected skin was treated 20 h post-infection with a single dose of either 200 μg or 300 μg of PlyPa03.	A 300 μg dose led to more than a 2-log reduction in bacterial load.	Raz et al. [46]
PyS2-GN4	*P. aeruginosa*	Mouse infection model	Bacteremia	PyS2-GN4 was injected intraperitoneally at 2.5, 5, 12.5, and 25 mg/kg 3 h after infection.	Protection rates were 73%, 80%, 93%, and 100% for 2.5, 5, 12.5, and 25 mg/kg, respectively.	Heselpoth et al. [118]
ClyJ	*S. pneumoniae*	Mouse infection model	Bacteremia	Single intraperitoneal doses: 0.3 mg/mouse and 0.4 mg/mouse at 1 h post-infection; 0.4 mg/mouse and 0.8 mg/mouse at 3 h post-infection; 1 mg/mouse tested for toxicity.	Dose-dependent protection: 90% (9/10) and 100% (10/10) survival at 10 days for 1 h post-infection doses. Lower survival (20%–50%) at 3 h post-infection. No toxicity observed at 1 mg/mouse.	Yang et al. [119]
LysGH15	*S. epidermidis*	Mouse infection model	Bacteremia	Mice were treated 1 h post-infection with LysGH15 at 5, 10, or 20 μg/mouse.	Complete recovery in all mice treated with 20 μg/mouse; significant reduction in bacterial load.	Zhang et al.[120]
LysAB2	*A. baumannii*	Mouse infection model	Bacteremia & intraperitoneal infection	Intraperitoneal infection model: single 100 μM/mouse (3.7 mg/kg) dose at 1 h post-infection. Bacteremia model: 32 μM/mouse (1.2 mg/kg) at 1 h, 200 μM/mouse (7.5 mg/kg) at 3 h post-infection.	In intraperitoneal infection, bacterial load decreased by 13-fold in ascites and 27-fold in blood at 4 h post-infection. 60% survival in mice with lethal *A. baumannii* bacteremia.	Peng et al. [121]
ClyR	*S. mutans*	Mouse dental colonization model	Dental caries	6 days post-inoculation, mice received oral ClyR (100 μl of 50 μg/ml) in 10 equal portions once daily for 3 weeks.	Significant reduction in *S. pyogenes* burden throughout the 5-week experiment, with a ~ 1.5-log reduction lasting ~2 weeks after treatment ended.	Yang et al. [122]
P307SQ-8C	*A. baumannii*	Mouse infection model	Skin infections	Bacteria colonized for 16–18 h before treatment with 200 μg P307SQ-8C.	Bacterial burden reduced by ~ 2 logs within 2 h.	Thandar et al. [92]
Ply30	*S. suis* & *S. equi subsp.*	Mouse infection model	Systemic	Intravenous injection of 2 mg Ply30 in 0.2 ml at 1 h post-infection (following intraperitoneal pathogen challenge).	Survival rate: *S. equi* subsp. (90%; 9/10) and *S. suis* (80%; 8/10) within 96 h.	Tang et al. [123]
Ply187	*S. aureus*	Mouse infection model	Endophthalmitis	Chimeric Ply187 (1 μg/μl) diluted in EB, administered intravitreally at 6 h (group I) or 12 h (group II) post-infection.	Eye retention rate: 90–95%. Significant bacterial load reduction and suppression of inflammatory markers (IL-6, IL-1β, TNF-α, MIP-2, KC).	Singh et al. [105]
PlyPy	*S. pyogenes*	Mouse infection model	Bacteremia	Single intraperitoneal dose of 0.25 mg or 0.50 mg PlyPy hemolysin administered 3 h post-infection.	Significant protection against mortality (log-rank test, *p* < 0.0001). Survival rates: 90% (18/20) at 0.25 mg, 95% (19/20) at 0.50 mg over 7 days.	Lood et al. [124]
PlySK1249	*S. agalactiae*	Mouse infection model	Bacteremia	Series 1: Single 22.5 mg/kg intraperitoneal dose at 1 h post-infection. Series 2: 45 mg/kg intraperitoneally at 2, 20, and 24 h post-infection.	Series 1: Improved survival at 48 and 72 h, but not significant. Series 2: Multi-dose treatment significantly improved survival (80%; 8/10 at 5 days).	Oechslin et al. [125]
Cpl-1	*S. pneumoniae*	Mouse infection model	Bacteremia	Intravenous treatment with daptomycin (0.4 mg/kg) or Cpl-1 (0.4 mg/kg or 1 mg/kg) alone or in combination, administered 1 h post-infection.	Simultaneous injections of daptomycin and Cpl-1, with sub-doses of each drug, significantly increased survival to 80% on day 7.	Vouillamoz et al. [126]
PlySs2	MRSA	Mouse infection model	Bacteremia	Intraperitoneal injection of 2 to 4 mg/ml PlySs2 3 h after infection.	Survival: MRSA-only (89%; 16/18), S. pyogenes-only (94%; 15/16), mixed (92%; 22/24).	Gilmer et al. [127]
ClyS	*S. aureus*	Mouse infection model	Skin infections	Topical application of 1%, 6%, or 10% (wt/wt) ClyS at 24 h post-implantation.	6% ClyS yielded a 2-log bacterial reduction; 10% resulted in a 3-log reduction compared to Aquaphor control.	Pastagia et al. [71]
LysGH15	MRSA	Mouse infection model	Bacteremia	Single i.p. injection of 0, 5, 10, 50, or 100 μg at 1 h post-infection; an additional regimen of 50 μg at 1, 2, 3, and 4 h post-infection in a separate experiment.	A single 50 μg dose at 1 h post-infection (at double the minimum lethal dose) provided significant protection (*p* < 0.01).	Gu et al. [128]
CHAPk	*S. aureus*	Mouse infection model	Nares	1 h post-infection, administer 925 μg/60 μl CHAPk (delivered as 20 μl per nostril and 20 μl orally per mouse).	A single treatment reduced nasal *S. aureus* Xen29 by 2 logs within 1 h.	Fenton et al. [129]
SAL200	MRSA	Mouse infection model	Bacteremia	Intravenous injection of SAL200 at doses of 12.5 mg/kg or 25 mg/kg, administered at 1, 25, and 49 h post-infection.	Prolonged survival and significant reduction in bacterial counts in blood and spleen.	Jun et al. [130]
CF-296	*S. aureus*	Mouse infection model	Thigh Infection	Single intravenous dose (0.5, 2.5, 5, 25, or 50 mg/kg) administered over 24 h.	CF-296 demonstrated a strong antibacterial dose-response, achieving a ≥ 1 log_10_ CFU/thigh reduction across most doses.	Asempa et al. [131]
Exebacase	*S. aureus*	Neutropenic murine model	Thigh Infection	2 h post-inoculation, subcutaneous injection of daptomycin plus six increasing doses of Exebacase (15–90 mg/kg).	Co-administration with daptomycin led to a mean log_10_ CFU/thigh reduction of −1.03 ± 0.72 (range: −0.77 ± 0.98 to −1.20 ± 0.59) across evaluated doses.	Asempa et al. [132]
CF-301	MRSA	Mouse infection model	Bacteremia	3 h post-inoculation, single intraperitoneal injection of CF-301 (0.25–5 mg/kg).	CF-301 enhanced survival in bacteremia by reducing blood MRSA 100-fold within 1 h.	Schuch et al.[26]
Exebacase	MRSA	Rabbit infection model	Infective endocarditis	24 h post-induction of IE, a single slow bolus dose of Exebacase (11 mg/kg i.v.) combined with daptomycin on the first treatment day.	MRSA counts in the combination group were significantly lower than untreated controls (*p* < 0.0001) and the daptomycin-alone group (*p* < 0.0001).	Shah et al. [133]
Exebacase	*S. aureus*	Human	Endocarditis	Clinical trial (NCT03163446): Day 14 responder rates were compared between Exebacase + antibiotics vs. antibiotics alone.	On day 14, response rates were 70.4% and 60.0%, respectively. In U.S. MRSA patients, median hospital stay was 4 days shorter, and 30-day readmission rates were 48% lower in the Exebacase-treated group.	Fowler et al. [28]
Exebacase	*S. aureus*	Mouse infection model	Lung	Exebacase (5 mg/kg i.v.) ± daptomycin (50 mg/kg s.c.), administered once daily for 3 days, starting 4 h post-intranasal infection.	At 14 days, survival rates were 50% (Exebacase alone) and 70% (Exebacase + daptomycin).	Swift et al. [134]
LSVT-1701	*Staphylococcal*	Healthy Volunteers	Health	Clinical trial (NCT03446053): Single-/multiple-dose study in healthy subjects.	Safe and well tolerated; exposure increased more than dose-proportionally without accumulation; 97% of TEAEs were mild.	Wire et al. [135]
Exebacase	*S. aureus*	Human	Bacteremia & endocarditis	Clinical trial (NCT04160468): Comparative study of Exebacase plus antibiotics versus antibiotics alone in MRSA infections.	Exebacase combined with antibiotics did not improve the clinical response at day 14 compared to antibiotics alone.	Fowler et al. [31]

### Animal models of infection

The selection of an appropriate animal infection model is critical for evaluating the in vivo efficacy of lysins. Various animal models have been developed to simulate infections including bacteremia, pneumonia, endophthalmitis, and keratitis. Most studies have primarily focused on systemic infections, with murine models being the most commonly employed, followed by *Galleria mellonella* larvae and zebrafish. These models effectively mimic clinical infections and provide reliable insights into the therapeutic potential of lysins. As experimental demands grow and research advances, larger animal models have also been adopted to further assess lysin efficacy. For instance, Shah et al. [133] and Huang et al. [113] utilized a rabbit model of infective endocarditis to demonstrate the therapeutic potential of lysins, both alone and in combination with daptomycin. Wang et al. [117] investigated the application of lysin in porcine streptococcal infections using a piglet model. Notably, lysin has also shown promising efficacy in topical wound and skin infection models. Euler et al. [111] reported that a single dose of PlyKp104 significantly reduced *Streptococcus pneumoniae* burden by more than two orders of magnitude in a murine skin infection model, highlighting its potential as a topical antimicrobial agent.

### Drug delivery programs

After identifying a suitable animal model, selecting an appropriate dosing regimen is essential for the success of lysin-based therapy. As a small-molecule protein drug, lysin is typically administered systemically to exert its antimicrobial effects. Common routes include intraperitoneal (i.p.), intravenous (i.v.), subcutaneous (s.c.), and intramuscular (i.m.) injections. However, these methods are often inefficient and may induce immune responses, potentially leading to therapeutic failure-one of the major challenges limiting the clinical development of lysin-based therapeutics [136]. To improve efficacy and reduce immunogenicity, alternative delivery strategies have been explored. For example, intratracheal administration of lysin Cpl-1 and intranasal delivery of lysin SAL200 have demonstrated significant reductions in bacterial loads of *S. pneumoniae* and *S. aureus*, respectively, in murine models [137,138]. Wang et al. developed and characterized a dry powder formulation of lysin Cpl-1, demonstrating that its bioactivity was retained after spray-drying. Formulations containing leucine combined with lactose or alginate exhibited favorable physicochemical properties (e.g. particle size, crystallinity, and hygroscopicity) and excellent aerosol performance, with fine particle fraction values exceeding 65%, making them promising candidates for inhalation-based treatment of pulmonary infections [82]. The timing and dosage of lysin administration are also critical factors influencing therapeutic outcomes. For instance, in a mouse bacteremia model, different doses of LysGH15 (5, 10, and 20 μg/mouse) were tested, and the highest dose (20 μg/mouse) resulted in complete bacterial clearance and full recovery [120]. Indiani et al. evaluated the synergistic effect of CF-301 with daptomycin in a rabbit infective endocarditis model, demonstrating enhanced efficacy when lysin dosage was appropriately adjusted [139]. Similarly, Singh et al. employed the C57BL/6 mouse model of *S. aureus* endophthalmitis to assess the impact of administration timing. Chimeric Ply187 was injected intravitreally at 6-hour and 12-hour post-infection, and mice were subsequently monitored for changes in inflammatory cytokines and chemokines. Notably, the 6-hour treatment group exhibited a significantly greater reduction in MIP-2 levels compared to the 12-hour group [105]. In certain cases, continuous or periodic administration may be necessary to maintain effective lysin concentrations. Therefore, optimal dosing regimens should be tailored according to infection type, disease progression, and host immune response to maximize therapeutic efficacy.

### Effectiveness

The effectiveness of lysin has been demonstrated across various animal infection models following the determination of appropriate dosing regimens. Lysin treatment has been shown to reduce bacterial burden, improve survival rates, slow disease progression, and enhance overall prognosis. For instance, in a murine bacteremia model, treatment with AVPL at doses of 300, 150, and 50 μg/mouse resulted in survival rates of 100%, 70%, and 10%, respectively, at 7 days post-infection, with survival correlating positively with dosage [69]. In a separate endophthalmitis model, bacterial counts in the PlyB-treated group were reduced by 5 log_10_ within 4 hours of treatment. Similarly, in a keratitis model, seven hours post-infection, *Bacillus cereus* CFU count in the eyes of PlyB-treated mice decreased by over 4 log_10_ compared to the untreated group [110]. Additionally, Wang et al. demonstrated that administration of Ply1228 (200 μg/mouse) one hour after bacterial challenge via intraperitoneal injection significantly reduced bacterial loads in the blood and major organs (liver, spleen, lungs, kidneys), while also markedly alleviating the inflammatory response and histopathological damage in infected mice [112].

The previously discussed strategies for lysin application, particularly in combination with antibiotics (Section 4.4), represent the most widely studied and promising approach in both animal experiments and potential future applications. Lysin-antibiotic combinations have demonstrated that they not only reduce the required antibiotic dose but also significantly enhance overall therapeutic efficacy [96]. Vouillamoz et al. were the first to report that co-administration of daptomycin (0.4 mg/kg) and Cpl-1 (0.4 mg/kg) markedly improved the 7-day survival rate of mice with pneumococcal bacteremia to 80%, compared to 0% in the untreated group and 35% and 0% in the monotherapy groups receiving daptomycin or Cpl-1 alone, respectively [126]. This synergy provides valuable insights and potential strategies for future lysin-antibiotic combinations. However, the results of phase III clinical trial NCT04160468, reported by Fowler et al., indicated that the combination of Exebacase and antibiotics failed to improve the clinical response at day 14 in patients with MRSA bacteremia/endocarditis. This outcome was inconsistent with the findings from the phase II trial. Several factors may explain this discrepancy. First, the observed efficacy in the phase II study may have been influenced by randomization bias. Second, this inconsistency underscores the need for cautious interpretation of clinical endpoints, as persistent symptoms can occur early in the disease course or may remain even after infection resolution. Furthermore, the appropriate selection of trial endpoints and threshold criteria remains crucial in determining clinical success [31].

### Safety

In animal studies, safety evaluations typically involve monitoring levels of inflammatory cytokines and chemokines, as these markers tend to increase during bacterial infections. However, lysin has been shown to effectively reduce these inflammatory markers [105]. This suggests that lysin may mitigate the body’s immune response by decreasing both the bacterial load and virulence of pathogenic bacteria.

Yang et al. found that repeated oral administration of the lysin ClyR induced a detectable immune response in mouse serum. However, no ClyR-specific antibodies were detected in mouse saliva, indicating that oral delivery of ClyR may avoid the induction of potentially neutralizing antibodies [122]. Although extensive animal studies have been conducted, conventional animal models are not entirely reliable for assessing human immunogenicity [140]. Therefore, in addition to evaluating safety and efficacy in animal models, further large-scale mammalian studies and clinical trials are essential to fully assess the safety and clinical applicability of lysin.

In a clinical study, Fowler et al. reported findings from trial NCT03163446 that assessed Exebacase for treating *S. aureus* bloodstream infections and endocarditis. Results demonstrated clinical response rates of 70.4% and 60.0% at day 14 in the Exebacase-antibiotic combination group and the antibiotic-alone group, respectively, with similar rates of adverse events (AEs) in both groups. Notably, the Exebacase-treated group had a 4-day shorter median hospital stay and a 48% lower 30-day readmission rate compared to those receiving antibiotics alone [28]. In a separate study, Wire et al. evaluated the safety and pharmacokinetics of LSVT-1701 in healthy adults, demonstrating dose-proportional drug exposure, no accumulation, and predominantly mild adverse events [135]. While Echterhof et al. examined the in vivo pharmacokinetics of neutrophil responses during phagocytosis [141], lysin-specific pharmacokinetic properties remain largely underexplored.

Overall, animal studies have demonstrated the promising therapeutic potential of lysins, particularly against Gram-positive pathogens such as *S. aureus*, and related clinical trials are progressing in an orderly manner. However, clinical data regarding the efficacy of lysins against Gram-negative bacteria remain limited. Future research will need to focus on expanding and analyzing clinical datasets to further validate the efficacy and clinical applicability of lysin-based therapies.

## Challenges and prospects

Throughout the development of antibacterial therapies, it has become evident that every agent possesses inherent advantages and limitations. While lysin exhibits unique advantages and promising applications, it still faces several critical challenges in research and clinical applications as a protein-based antimicrobial agent. These challenges primarily include the following aspects [1]: difficulties in the isolation and characterization of natural lysins [2]; challenges in heterologous expression and low production yields [3]; short biological half-life and limited understanding of optimal delivery mechanisms, owing to their exogenous protein nature; and [4] the lack of standardized in vivo therapeutic protocols and comprehensive efficacy evaluations. In the following sections, the following sections discuss each of these challenges in detail and propose practical solutions based on existing literature.

### Challenges in annotating and identifying natural lysins

Lysins have garnered significant attention due to their modular architecture and several advantages, including relative safety, high efficiency, specificity, the ability to degrade bacterial cell walls, and a low risk of inducing drug resistance. The application strategies summarized in the previous section rely heavily on experimentally validated lysins, which in turn rely on genome sequencing and subsequent functional annotation for their identification. However, a substantial portion of genomic sequencing data remains unannotated and cannot be assigned to a known organism or function, often referred to as genomic “dark matter.” Consequently, less than 2% of lysins have been identified and experimentally characterized to date, representing only a small subset of those potentially present in nature. This severely restricts the scope of lysin discovery and its broader therapeutic application.

With the rapid advancement of artificial intelligence (AI) and machine learning in recent years, novel approaches have emerged for the isolation, screening, and identification of lysins. Fu et al. proposed a convolutional neural network (CNN)-based framework, DeepMineLys, which achieved an F1 score of 84.00%, surpassing existing methods by 20.84% when validated with an independent dataset. The effectiveness of DeepMineLys was further confirmed through experimental validation of drug candidates, and the framework is also applicable to the discovery of other proteins [142]. Notably, Zhang et al. developed DeepLysin, the first AI-driven platform engineered for high-throughput screening of large-molecule antibacterial proteins, such as novel lysins, from the uncharacterized genomic data. Using DeepLysin, putative lysins were screened from uncharacterized *S. aureus* phages, and 17 novel lysins were randomly selected for experimental validation. Among these, seven lysins demonstrated potent in vitro antibacterial activity. Particularly, when compared with existing lysins, such as the currently phase III clinical lysin CF-301, LLysSA9 demonstrated more outstanding efficacy. The remarkable therapeutic potential of LLysSA9 was further validated in mouse models of bacteremia and wound infections. This further confirmed the accuracy and application potential of the DeepLysin platform [143]. These studies demonstrate the combination of computer methods, such as artificial intelligence and experimentation, to speed up the identification of novel lysins.

### Low expression yield of lysin

Currently, lysins are primarily expressed in prokaryotic systems, particularly *E. coli*, using genetic engineering techniques. However, the need for affinity chromatography and endotoxin removal significantly increases production costs. Optimizing expression strategies can not only improve yield but also enhance economic feasibility, thereby supporting the broader industrial application of lysins.

The selection of traditional eukaryotic expression systems, such as Gram-positive bacteria (e.g. *Bacillus subtilis* or *Lactobacillus*), yeasts, or cellular systems, for scale-up production and the refinement of the purification process after large-scale production of lysins can help reduce costs at the production stage. Additionally, emerging platforms such as green microalgae [144], plant virus-based expression systems [145], and edible plants [146] have demonstrated the capability to express and produce phage lysins while maintaining consistent antimicrobial activity.

When using plants as an expression system, expensive bioreactors and culture media are not required, thus minimizing the loss of consumables. The production cost of antimicrobial proteins in plant-based systems ranges from $3.00 to $6.88 per gram [147], providing a cost-effective strategy for the industrial production of phage lysins. Cremelie et al. offered a comprehensive review of available expression systems for phage lysin production in their article [148].

### Short half-life, easy degradation, and difficult delivery of lysin

Despite the short time required for lysin to act, its half-life in vivo is relatively short due to protease activity and immune clearance. Reported half-lives range from 20.5 [149], 22.5 [150] to 60 minutes [151]. Furthermore, when administered orally or enterally, lysins are easily degraded by gastric acid and proteases, resulting in reduced bioavailability and irreversible structural damage [152]. Lysin stability is influenced by factors such as temperature, pH, and ionic strength, with many lysins prone to precipitation at physiological temperatures. Therefore, future selection and engineering of lysins should account for specific application conditions, and their efficacy should be evaluated under relevant environmental parameters. Vázquez et al. also pointed out that the bactericidal effect of phage lysins largely depends on the buffer tension and ionic strength. For experimental consistency, it is essential to use the same incubation buffer as the diluent for post-serial dilution and inoculation [153].

To address the challenges of lysin delivery, several modification strategies are being explored, including directed evolution, chimeric recombination, site-directed mutagenesis, and heterologous fusion to optimize recombinant phage lysins. Additionally, molecular docking and molecular dynamics simulations have become powerful tools for lysin modification, and the ongoing resolution of lysin structures and sequences makes computer simulations for pre-mutation and modification a promising option [154]. To overcome delivery barriers, various approaches, such as liposome or nanoparticle encapsulation, and fusion with bacteriocins, are being investigated to enable effective sterilization of lysins under different environmental conditions [155]. The delivery of phages and lysins for clinical applications has also been extensively studied [156].

Despite the broad application prospects of the aforementioned engineering and delivery strategies, multiple challenges remain. Protein engineering techniques such as directed evolution and site-directed mutagenesis can improve the stability and activity of lysins; however, technical bottlenecks in mutant library construction, screening efficiency, and expression system compatibility continue to limit large-scale production and clinical translation. Meanwhile, delivery systems such as nanoparticles and liposomes still require optimization in terms of in vivo stability, targeting specificity, and immunogenicity control, and their high manufacturing costs hinder large-scale implementation. Overall, although technological advances offer new avenues for lysin application, successful clinical translation will require interdisciplinary collaboration to overcome these critical challenges.

### Lysin in vivo application protocols and efficacy are unclear

Preclinical studies in animal models and early-phase clinical trials have shown that lysins exhibit promising antibacterial activity, making them attractive candidates for the treatment of various infectious diseases. As a protein, lysin is closer to traditional antimicrobial drugs than phages, aligning more closely with the existing approval and management processes for antimicrobial agents. This is one of the primary reasons why the industry is inclined to develop lysin. However, significant challenges remain before lysins can be established as routine therapeutic agents, particularly in the context of long-term systemic administration.

Firstly, there is limited data on the interaction of lysin with the human body [157]. Available data, especially for enzymes targeting Gram-negative bacteria (such as *Pseudomonas*, *Klebsiella*, and *Fusobacterium*), are mainly confined to efficacy studies in animal models [77,158]. A critical prerequisite for the clinical translation of lysins is the development of a comprehensive pharmacological evaluation system. This should include investigations into pharmacokinetics, host immune responses, and pharmacodynamics against pathogenic bacteria. The safety of lysin therapy can be further validated by determining the minimum bactericidal concentration and the required dosage for different pathogens. Currently, some researchers are working to address the limitations of systemic lysin administration, such as immunogenicity, short half-life, and intracellular targeting.

Fortunately, notable progress has been made in the clinical use of both phages and lysins, with the World Medical Association permitting their use through its Declaration of Helsinki (§37) [27]. In parallel, some countries and regions have issued interim regulations that allow the use of unconventional therapies in life-threatening situations, particularly when all conventional treatments have failed (FDA Expanded Access Program Report, 2020). In conclusion, further research is essential to overcome current limitations and support regulatory approval for lysin-based therapies, thereby contributing to global efforts in combating the escalating crisis of antimicrobial resistance.

### Barriers and insights in the clinical translation of lysins

As the first phage lysin candidate to enter Phase III clinical trials, Exebacase has drawn significant attention for its potential in treating *S. aureus* infections, particularly MRSA. However, the DISRUPT trial did not achieve statistical significance for its pre-specified primary endpoint – day-14 clinical response rate in MRSA-infected patients (50.0% vs 60.6%, *p* = 0.392) – despite showing a trend toward clinical improvement in the modified ITT (mITT) population (70.4% vs 60.0%, *p* = 0.078) [31]. These results reflect systemic challenges in translating lysins from the laboratory to clinical application, including target population selection, sensitivity of endpoints, and optimization of dosing strategies [28].

From a pharmacological perspective, although Exebacase exhibits potent in vitro lytic activity and low risk of resistance development [159], its status as an exogenous protein results in a short in vivo half-life and susceptibility to proteolytic degradation [160]. A single intravenous dose may be insufficient to maintain effective concentrations at the infection site [28], and its activity depends on host serum cofactors such as lysozyme and albumin, which differ significantly between animal models and humans – leading to possible misestimation of efficacy in preclinical studies [133,139]. The DISRUPT trial design also raised concerns: a single-dose regimen and the use of a day-14 clinical response as the primary endpoint may not have adequately captured the rapid and transient bactericidal action characteristic of lysins [161]. In particular, observed trends of benefit in the MRSA subgroup suggest that an enrichment strategy or more sensitive microbiological endpoints (e.g. time to blood culture clearance) might have better highlighted clinical advantages.

Although Exebacase did not elicit serious immunogenic adverse events in clinical trials [28,162], the potential for neutralizing antibody formation during long-term administration remains a concern that warrants further investigation [159]. Importantly, synergy with conventional antibiotics constitutes a core advantage of lysins. Preclinical data have demonstrated that Exebacase significantly enhances the efficacy of various antibiotics, including daptomycin, both in vitro and in animal models [132,134,162]. However, in the Phase III DISRUPT clinical trial, when used as an adjunctive therapy alongside standard-of-care antibiotics (including but not limited to vancomycin), this synergy did not translate into a statistically significant clinical benefit [31]. This discrepancy may be attributed to heterogeneity in the host microenvironment (such as differential drug penetration at infection sites) or the choice of antibiotic combinations [96]. Therefore, future studies should focus on optimizing combination strategies, including antibiotic selection, dosing schedules, and therapeutic windows.

From a regulatory standpoint, lysins face a lack of standardized evaluation frameworks. Traditional antibiotic susceptibility testing methods (e.g. MIC) may not be applicable [30,159], and the correlation between microbiological and clinical endpoints needs reevaluation. The case of Exebacase exemplifies the translational gap between mechanistic promise and clinical validation, underscoring the need for systemic reform in the design, assessment, and regulation of novel antimicrobial therapies.

Despite the Phase III setback, Exebacase provides a strong foundation for further development. Strategies such as PEGylation to prolong half-life, nanocarrier delivery systems to enhance targeting, or local administration (e.g. for pulmonary infections) may help overcome current limitations [134]. Ultimately, the successful clinical implementation of lysins will depend on an integrated, multidisciplinary approach that combines molecular engineering, targeted delivery systems, and adaptive clinical trial designs – thereby unlocking their full therapeutic potential amid the escalating threat of antibiotic resistance.

## Concluding remarks

In recent years, the prevalence of MDR bacteria has surged, while the development of novel antibiotics has struggled to keep pace with the threat posed by these resistant pathogens. In the post-antibiotic era, phage lysins have emerged as promising antibacterial agents with the potential to address this pressing challenge. Lysin, a functional protein of natural origin with a modular structure, efficiently and specifically cleaves peptidoglycan in bacterial cell walls. Studies have shown that lysin exerts strong antibacterial effects against Gram-positive bacteria both in vivo and in vitro. Their rapid and targeted lytic activity allows them to be used in combination with antibiotics to enhance their efficacy, regardless of antibiotic resistance. Lysin also works synergistically with OMPs, antimicrobial peptides, and phages, demonstrating broad complementary effects across different therapeutic strategies. Furthermore, lysins exhibit a broader lytic spectrum than their parental phages, and no bacterial resistance to lysins has been reported to date. In preclinical studies, lysins have shown favorable safety profiles and low immunogenicity. Due to their high modularity, they can be engineered to optimize physicochemical properties and therapeutic performance.

Although lysin offers many advantages, its large-scale clinical application still faces several challenges, including concerns regarding its efficacy, safety, pharmacokinetics, dosing regimen, and regulatory approval, all of which require further investigation. To advance the clinical translation of lysin, it is crucial to explore novel lysins by integrating artificial intelligence and machine learning, as well as to conduct in-depth studies on the molecular dynamics of lysins in vivo and the mechanisms behind lysin cleavage. At the same time, it is essential to explore large-scale production processes for lysins, evaluate their druggability, and advance the establishment of drug approval standards worldwide. Through these efforts, lysin is expected to transcend its current limitations against Gram-positive bacteria and extend its spectrum to target MDR pathogens. Although lysin is still some way from becoming a mainstream antimicrobial agent, we are confident that, with the resolution of these challenges, phage lysin will become a significant breakthrough in the development of novel antimicrobial drugs.

## Data Availability

Data sharing is not applicable to this article as no new data were created or analyzed in this study.
